# Biosolids as Safe Fertilizers for Soybean and Maize: Enhanced Nutrition Without Antibiotic Residues or Phenotypic Resistance in Grains

**DOI:** 10.3390/antibiotics14121244

**Published:** 2025-12-09

**Authors:** Thiago Nery Menezes, Keite Silva Nogueira, Ruanita Veiga, Raizza Zorman Marques, André Carlos Auler, Leandro Flávio Carneiro, Murilo Duma, Rebert Skalisz, Marcelo Pedrosa Gomes

**Affiliations:** 1 Departamento de Patologia Básica, Setor de Ciências Biológicas, Universidade Federal do Paraná, Avenida Coronel Francisco H. dos Santos, 100, Centro Politécnico Jardim das Américas, Curitiba 81531-980, Paraná, Brazilkeite.nogueira@ufpr.br (K.S.N.);; 2 Laboratório de Bacteriologia, Complexo Hospital de Clínicas, Universidade Federal do Paraná, Rua Padre Camargo, 280, Curitiba 80060-240, Paraná, Brazil; 3 Laboratório de Fisiologia de Plantas sob Estresse, Departamento de Botânica, Setor de Ciências Biológicas, Universidade Federal do Paraná, Avenida Coronel Francisco H. dos Santos, 100, Centro Politécnico Jardim das Américas, Curitiba 81531-980, Paraná, Brazil; 4 Departamento de Solos e Engenharia Agrícola, Universidade Federal do Paraná, Rua dos Funcionários, Curitiba 80035-050, Paraná, Brazil; 5 Departamento de Fitotecnia e Fitosanidade, Universidade Federal do Paraná, Rua dos Funcionários, 1540, Curitiba 80035-050, Paraná, Brazil; 6 Companhia de Saneamento do Paraná (SANEPAR)—Sede Administrativa, Rua Engenheiros Rebouças, 1376, Rebouças, Curitiba 80215-900, Paraná, Brazil

**Keywords:** antimicrobial resistance, biosolids, rhizosphere, antibiotics, fluoroquinolones

## Abstract

**Background/Objective:** Sewage sludge (biosolids) is increasingly reused as a fertilizer to recycle nutrients and close material cycles; however, concerns persist regarding antibiotics and antimicrobial resistance. This study evaluated the agronomic safety and microbiological integrity of biosolid fertilization in soybean and maize systems, with particular attention to grain quality and food safety. **Methods:** Soybean and maize were cultivated in greenhouse microcosms under biosolid or mineral fertilization. Soil, roots, shoots, and grains were analyzed for antibiotic residues using LC–MS/MS and antibiotic-resistant bacteria (ARB) using culture-based assays. Minimum inhibitory concentrations for isolates from grains were compared with clinical breakpoints to verify phenotypic susceptibility. Multivariate analyses (PCA) integrating real-time antibiotic concentrations and updated resistance indicators were performed using centered and scaled data. **Results:** Fluoroquinolones were the predominant residues introduced by biosolids and exhibited consistent time-dependent declines across all treatments, although low concentrations remained detectable at 90 d in several soil–fertilizer–crop combinations. Tetracyclines, macrolides, and sulfonamides showed similar decreasing trends, with planted soils displaying faster dissipation than bulk control soils. Biosolid fertilization increased shoot biomass by 1.5–2.3-fold and nitrogen, phosphorus, and potassium uptake by 30–60% without impairing soybean nodulation or nitrogenase function. ARB was observed in all soils, including mineral and plant-free controls, indicating a natural background resistome. Ciprofloxacin-resistant isolates were detected in one simple sampling point, and MDR proportions were transient (67%), returning to their background levels by 45–90 days. PCA showed that crop presence, not fertilizer type, was the primary driver of microbial ordination, and that antibiotic concentrations and resistance indicators were only weakly aligned, indicating a limited selective pressure. No antibiotic residues or phenotypically resistant bacteria were detected in the soybean or maize grains. **Conclusions:** Updated residue, resistance, and multivariate data confirmed that biosolids did not induce, amplify, or transfer antibiotic resistance and maintained complete grain safety. Properly treated biosolids function as safe, agronomically beneficial fertilizers aligned with One Health goals, enhancing crop productivity without compromising food quality or increasing antimicrobial resistance.

## 1. Introduction

The application of sewage sludge (biosolids) in agriculture has emerged as a cornerstone of circular economy strategies, aiming to recycle nutrients back into soils and reduce the reliance on synthetic fertilizers. In addition to providing organic matter and essential macronutrients, biosolids can enhance soil fertility and crop productivity [1]. However, their use raises concerns regarding contaminants of emerging concern (CECs), including pharmaceuticals, personal care products, microplastics, heavy metals, pesticides, and antibiotic-resistant bacteria. These compounds may enter agroecosystems through sludge application and ultimately affect soil health, crop safety, and environmental quality [2,3]. In a global assessment, 229 of 419 CECs investigated were detected in biosolids [1]. These contaminants can accumulate in agricultural soils and be absorbed by crops, with leafy vegetables exhibiting the highest uptake potential, followed by root vegetables and cereals [4]. However, some studies have reported little or no contribution of biosolids to soil contamination with CECs [4,5]. For example, in field experiments in Québec, Charbonneau et al. [6] observed that municipal biosolids applied to soybean and maize fields contained measurable concentrations of glyphosate and its metabolite AMPA; however, their application did not increase soil contamination or reduce crop productivity. These findings highlight the dual role of biosolids as valuable nutrient sources and potential vectors of chemical residues, reinforcing the need to broaden this line of investigation to include additional classes of emerging contaminants.

Among these, antibiotics are a particularly urgent concern. Widely used in human and veterinary medicine, antibiotics are often incompletely removed during wastewater treatment, resulting in their accumulation in sludge [7]. In Chinese wastewater treatment plants (WWTPs), the average sludge concentration reached 186.8 μg/kg, with ciprofloxacin, sulfamethoxazole, erythromycin, azithromycin, and tetracycline posing moderate to high ecological risks [8]. Spanish WWTPs reported ciprofloxacin and levofloxacin as the most abundant compounds, with maximum concentrations of 623 and 893 ng/g, respectively [9]. In Poland, ciprofloxacin concentrations reached 28 μg/g and norfloxacin 5.3 μg/g [10], while smaller WWTPs elsewhere showed even higher antimicrobial levels, with tetracycline up to 900.24 μg/kg [11]. Conventional biological treatment processes exhibit limited efficiency in eliminating antibiotic-resistant bacteria (ARB). Elevated abundance of resistant *Escherichia coli* has been observed in activated sludge relative to influent wastewater, with resistance patterns varying across antibiotic classes [12]. Sludge consistently harbors multidrug-resistant bacteria, such as *Enterococcus*, *Citrobacter*, and *Klebsiella* [13,14]. Reported ARB abundance ranges from 10^3^ to 10^9^ CFU/g, with resistance to azithromycin and ciprofloxacin being particularly prevalent [15,16]. Clinically relevant strains, including extended-spectrum β-lactamase-producing organisms and vancomycin-resistant enterococci, persist through treatment processes and are found in the final effluents and biosolids [17]. Therefore, when applied to agricultural soils, residual antibiotics and ARB may alter microbial community dynamics, exert selective pressure, and promote the emergence and spread of antibiotic resistance. This process contributes to the global crisis of antimicrobial resistance (AMR), which is recognized by the World Health Organization as one of the most urgent threats to public health [18]. Despite this urgency, relatively few studies have directly assessed the occurrence of antibiotics and ARB in agroecosystems managed with biosolid fertilization.

The implications of antibiotic contamination extend beyond environmental microbiology and agronomic performance. In soybean, a legume that depends on symbiosis with *Bradyrhizobium* for biological nitrogen fixation, antibiotics in the soil may disrupt rhizobial survival, nodulation, and nodule activity, ultimately affecting plant nutrition and yield [19]. In maize, although no such symbiosis occurs, the persistence of ARB in soils and potential transfer into roots, shoots, or grains raises questions about possible transmission pathways from the field to the food chain [20]. Hence, evaluating the dual impact of biosolid application on crop performance and antibiotic resistance dynamics is crucial for assessing safety and sustainability. Despite substantial advances, there is a clear lack of integrated studies that simultaneously quantify antibiotic residues, assess phenotypic resistance patterns, and measure crop performance within the same experimental system. Most available research examines these components separately [16,21], limiting the capacity to determine whether nutrient benefits and AMR risks co-occur or whether biosolid fertilization can be optimized to maximize agronomic value while minimizing microbiological hazards. This gap is particularly relevant in tropical and subtropical agricultural contexts, where biosolid application is expanding, but empirical evidence of its safety remains limited. Here, we present a comprehensive greenhouse pot experiment designed to address this research gap. We compared soybean and maize cultivated under mineral and sewage sludge fertilization. We quantified antibiotic residues and resistant bacteria in soils, roots, shoots, and grains, while simultaneously assessing nodulation, biomass accumulation, and yield in soybean plants. By integrating agronomic, chemical, and microbiological endpoints within a single experimental framework, this study provides novel insights into whether sewage sludge can function as a sustainable fertilization strategy without exacerbating the risks associated with antibiotic resistance.

## 2. Results

### 2.1. Characterization of Sewage Sludge

**Figure 1:**
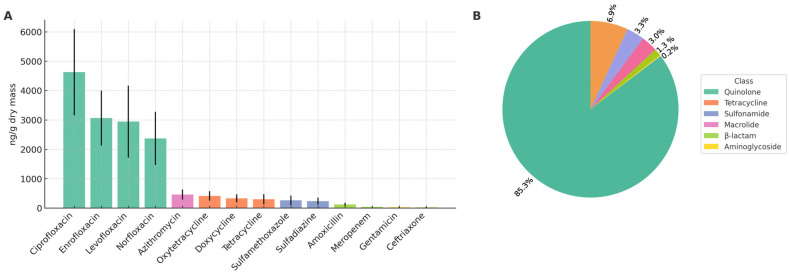
Antibiotic profile of sewage sludge. (**A**) Concentrations of individual compounds (ng/g dry mass). (**B**) Relative contribution of antibiotic classes to the total contaminant load in the environment.

The sewage sludge had an alkaline pH (12.36), high total solids (428,642 mg/kg), and total organic carbon content of 77,937 mg/kg (Table S1). The macronutrient concentrations were 14,495 mg/kg for nitrogen, 6961 mg/kg for phosphorus, and 706 mg/kg for potassium. Calcium and magnesium reached 115,275 mg/kg and 73,720 mg/kg, respectively, while sulfur was 14,198 mg/kg. Among the metals, zinc (1677 mg/kg) and copper (155 mg/kg) were the most abundant. Nickel (42 mg/kg), chromium (44 mg/kg), and lead (21 mg/kg) were detected at lower concentrations. Arsenic, cadmium, mercury, molybdenum, and selenium were below the quantification limits. Microbiological indicators showed <0.54 NMP g^−1^ ST for thermotolerant coliforms and <0.1 eggs g^−1^ ST for viable helminth eggs. The antibiotic profile (Figure 1) showed that fluoroquinolones were the predominant class, including ciprofloxacin, enrofloxacin, levofloxacin, and norfloxacin. Lower concentrations of macrolides, tetracyclines, sulfonamides, β-lactams, and aminoglycosides were also observed.

### 2.2. Agronomic Endpoints

**Figure 2:**
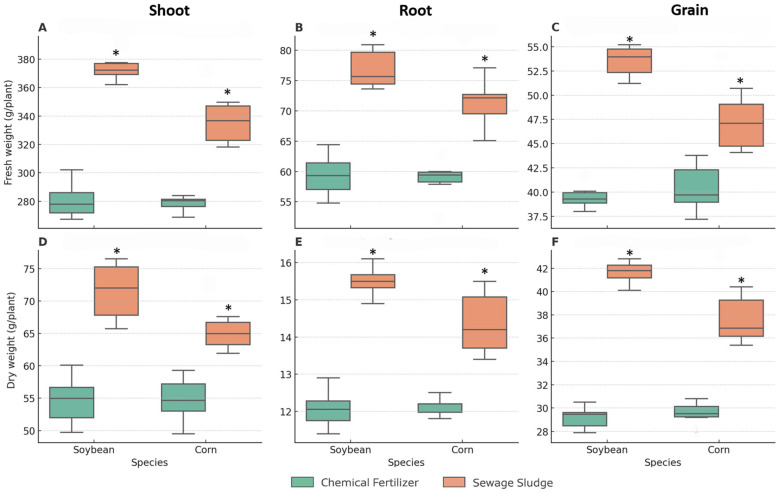
(**A**–**C**) Fresh and (**D**–**F**) dry weights of soybean and maize under mineral or sewage sludge fertilization. Boxplots represent the median, interquartile range, and whiskers (*n* = 5). Asterisks denote significant differences (*p* < 0.05, Tukey’s HSD test).

Biomass accumulation (fresh and dry weights) was consistently higher in the sewage sludge-fertilized plants than in those subjected to mineral fertilization (Figure 2). In soybean, sludge increased fresh weight by 25–30% across samplings, whereas in maize, the increase ranged from 15–20%. Dry weight showed a similar pattern, with statistically significant differences between the two crops (*p* < 0.05). Soybean nodulation parameters at 45 days did not differ among the fertilization treatments (Table S2). The total number of nodules, distribution across size classes, fresh and dry mass of nodules, percentage of pink nodules, and nitrogenase activity remained comparable between the sludge and mineral treatments.

### 2.3. Nutrient Uptake (N, P, K)

**Figure 3:**
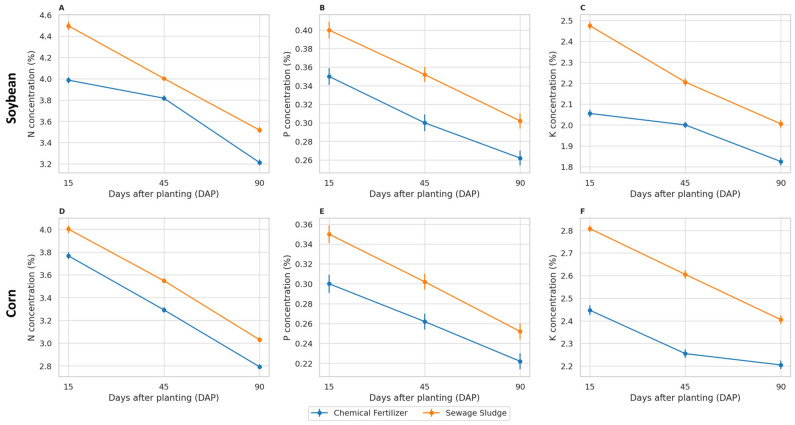
Nitrogen (**A**,**D**), phosphorus (**B**,**E**), and potassium (**C**,**F**) concentrations (%) in the shoots of soybean (**top row**) and maize (**bottom row**) under mineral or sewage sludge fertilization. Values are expressed as the mean ± SE (*n* = 5).

The concentrations of nitrogen, phosphorus, and potassium in the shoots decreased over time for both crops and fertilization treatments (Figure 3). At each sampling date (15, 45, and 90 days), plants cultivated with sewage sludge showed consistently higher N, P, and K concentrations than those with mineral fertilization. In soybean, two-way ANOVA indicated significant effects of treatment, time, and their interaction on N and K, and significant effects of treatment and time on P (Table S3). In maize, significant effects of treatment and time were observed for all nutrients, with a significant treatment × time interaction for P and K (Table S3).

### 2.4. Antibiotic Residues in Soil and Plant Tissues

**Figure 4:**
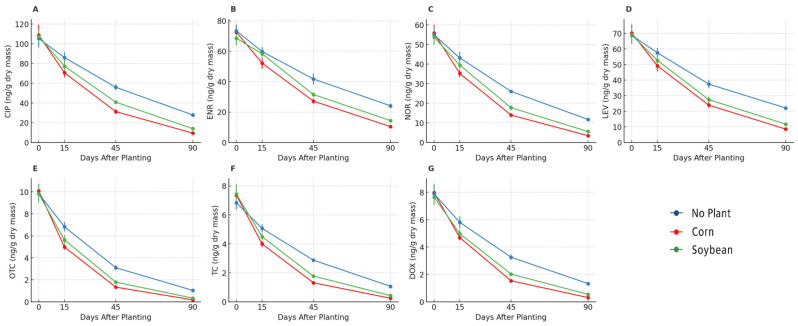
Dissipation of fluoroquinolones and tetracyclines in soils under sewage sludge fertilization. Temporal dynamics of ciprofloxacin (**A**), enrofloxacin (**B**), norfloxacin (**C**), levofloxacin (**D**), oxytetracycline (**E**), tetracycline (**F**), and doxycycline (**G**) in bulk soil (no plants), soybean rhizosphere, and maize rhizosphere. Values are expressed as the mean ± SE (*n* = 5).

**Figure 5:**
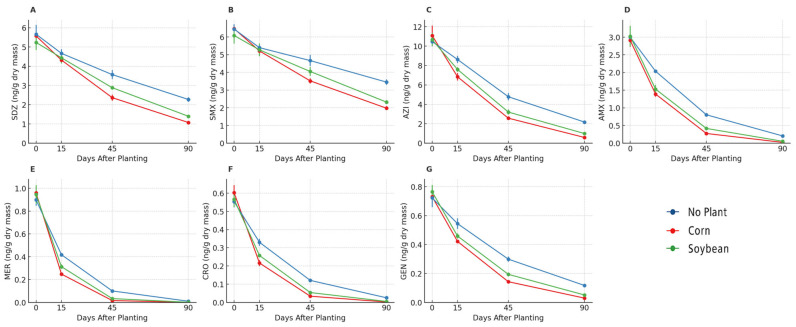
Dissipation of sulfonamides, macrolides, β-lactams, and aminoglycosides in soils under sewage sludge fertilization. Temporal dynamics of sulfadiazine (**A**), sulfamethoxazole (**B**), azithromycin (**C**), amoxicillin (**D**), meropenem (**E**), ceftriaxone (**F**), and gentamicin (**G**) in bulk soil (no plants), soybean rhizosphere, and maize rhizosphere. Values are expressed as the mean ± SE (*n* = 5).

Antibiotic residues were consistently detected in sludge-amended soils on day 0 and declined progressively until day 90 (Figure 4 and Figure 5). Among the fluoroquinolones, ciprofloxacin, enrofloxacin, norfloxacin, and levofloxacin showed the highest initial concentrations (60–120 ng/g dry mass) (Figure 4A–D). Dissipation was faster in planted soils than in bulk soils, with residues decreasing to <15 ng/g by day 90 in both soybean and maize rhizospheres, whereas bulk soils retained higher concentrations. In some cases (e.g., enrofloxacin and norfloxacin), maize rhizospheres showed slightly lower concentrations than soybean at intermediate times (days 45 and 90). Tetracyclines (oxytetracycline, tetracycline, and doxycycline) were detected at initial levels of 7–10 ng/g (Figure 4E–G). Concentrations dropped below detection limits by day 90 in both soybean and maize rhizospheres, whereas trace amounts persisted in bulk soils. Maize soils tended to reach non-detectable levels earlier (day 45 for doxycycline and tetracycline) than soybean soils, which still showed residual values at this time point. Sulfonamides (sulfadiazine and sulfamethoxazole) and the macrolide azithromycin were present at lower initial concentrations (≤12 ng/g) (Figure 5A–C). By day 90, these compounds were no longer detected in soybean and maize soils but remained measurable in bulk soils. The concentration profiles were similar between soybean and maize, with no consistent differences. β-lactams (amoxicillin, meropenem, ceftriaxone) and the aminoglycoside gentamicin were detected at sub-ng/g levels (≤1 ng/g) (Figure 5D–G). These compounds dissipated rapidly and were below the detection limit by 45–90 days in all treatments, including soybean, maize, and bulk soils.

**Figure 6:**
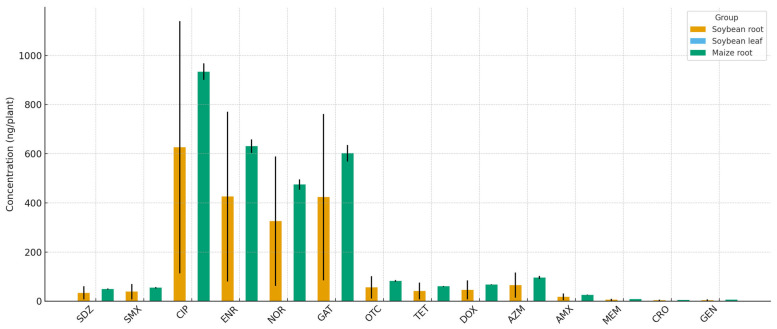
Antibiotic residues in soybean roots, soybean leaves, and maize roots after sludge fertilization. Bars represent the mean concentrations (ng/plant) ± SE (*n* = 5). Only compartments with detectable residues are shown; all other tissues had no detectable levels of residues.

Antibiotic residues were also detected in the roots and, to a lesser extent, in the shoots of soybean and maize, whereas grains from both crops showed no detectable levels of any compound (Figure 6). Fluoroquinolones were predominant in the roots. Ciprofloxacin, enrofloxacin, norfloxacin, and gatifloxacin reached concentrations of 300–950 ng/plant in soybean and maize roots. Lower concentrations (≤100 ng/plant) of tetracyclines (oxytetracycline, tetracycline, and doxycycline), sulfonamides (sulfadiazine and sulfamethoxazole), and the macrolide azithromycin were also detected. β-lactams (amoxicillin, ceftriaxone), meropenem, and gentamicin were either absent or present at trace levels near the quantification limit.

### 2.5. Antibiotic-Resistant Bacteria (ARB)

#### 2.5.1. Soil

ARB were consistently detected in soils under both mineral and sludge fertilization (Table 1 and Table S4). Resistance to third-generation cephalosporins (CTG_Resis._) and carbapenems (CARB_Resis._) occurred sporadically across all treatments and sampling times, with no consistent trend distinguishing the sludge from mineral fertilization. In contrast, ciprofloxacin-resistant isolates (CIPRO_Resis._) were detected at only one sampling point (15 days) in corn fertilized with sewage sludge, soybeans with chemical fertilizer and in soil with no plant and chemical fertilizer. The proportion of multidrug-resistant isolates (MDR; resistance to ≥2 antibiotics) was generally low across treatments, ranging from 0% to 25%, and was transient. The highest MDR proportion (67%) occurred in maize soils amended with fertilizer at 15 days; however, these increases did not persist, and all treatments converged to 0% MDR by 45–90 days, regardless of the crop or fertilization type. No sustained elevation of MDR frequency was associated with sludge application, and planted soils showed rapid normalization. The identified bacterial genera included Pseudomonas, Klebsiella, Serratia, Enterobacter, and Acinetobacter (Supplementary Table S4).

#### 2.5.2. Grains

Bacterial isolates were recovered from soybean and maize grains; however, the minimum inhibitory concentrations (MICs) for ceftazidime, ceftriaxone, meropenem, and ciprofloxacin were at or below the clinical breakpoints, indicating susceptibility rather than resistance. No multidrug resistance was observed in grains, and LC–MS/MS did not detect any antibiotic residues in grain tissues (Figure 6).

### 2.6. Multivariate Analyses

**Figure 7:**
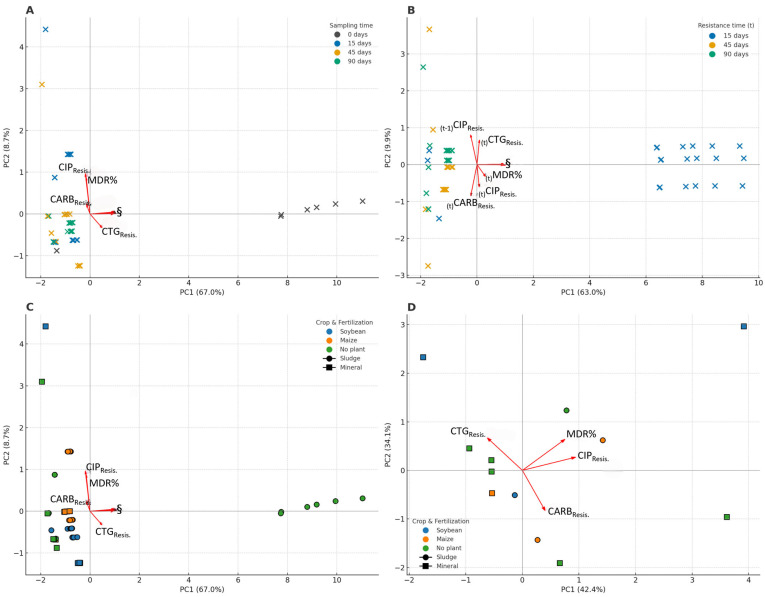
Multivariate analyses integrating soil antibiotic mixtures and bacterial resistance endpoints. (**A**) Principal Component Analysis (PCA) combining soil concentrations of all detected antibiotics (fluoroquinolones, tetracyclines, macrolides, sulfonamides, β-lactams, carbapenems, and aminoglycosides) with resistance endpoints (CTG_Resis._, CARB_Resis._, CIP_Resis._, and MDR%). Each point represents a soil sample, colored by the sampling time (0, 15, 45, and 90 days). The vectors indicate the contribution and direction of each antibiotic or resistance variable. Later sampling times tended to cluster along the positive PC1 axis due to shifts in microbial and resistance-related variables. Antibiotic concentrations decreased over time (Figure 4 and Figure 5), and their loadings were associated with early sampling dates. (**B**) Lagged-exposure PCA integrating antibiotic concentrations at time t − 1 with resistance measured at time t (15, 45, and 90 days). The points represent samples colored by resistance time t, and the vectors represent antibiotics at t − 1 and resistance at t and t − 1. The ordination highlights which antibiotic mixtures at the previous sampling step were most strongly aligned with the subsequent increase in CTG_Resis._, CARB_Resis._, CIP_Resis._, and MDR%. (**C**) PCA simultaneously integrating antibiotics, resistance endpoints, plant groups (soybean, maize, no-plant), and fertilization regimes (sludge vs. mineral). Points were coded by crop type and fertilization, whereas vectors represented antibiotic and resistance variables. The ordination showed clear structuring driven by plant presence, with rhizosphere soils clustering toward higher resistance gradients. (**D**) PCA using only resistance endpoints to visualize the effects of vegetation and fertilization on the resistance profile. The points are colored by crop and coded by fertilization type. The vectors illustrate the contribution of each resistance endpoint. Rhizosphere soils tended to be separated from bulk soils along the main resistance gradients, particularly CARB_Resis._ and CIP_Resis._ The symbol (§) marks positions where multiple vector labels overlap: in panels A and C, it indicates the cluster of antibiotic vectors CIP, ENR, LEV, NOR, AZI, OTC, TC, DOX, SMX, SDZ, AMX, MER, GEN, and CRO; in panel B, it indicates the overlapping vectors representing antibiotics at t − 1 together with resistance endpoints at t (CTG_Resis._, CARB_Resis._, CIP_Resis._, and MDR%), with the exception of _(t−1)_CIP_Resis._, which remains separately resolvable.

Across the full experiment, PCA integrating all antibiotics and resistance endpoints (Figure 7A) revealed a strong temporal gradient along PC1, which explained most variance. Samples from 45 and 90 days clustered toward the positive PC1 axis, coinciding with higher soil concentrations of fluoroquinolones, tetracyclines, sulfonamides and β-lactams. Resistance endpoints (CTG_Resis._, CARB_Resis._, CIP_Resis._) and MDR (%) aligned in the same direction, indicating that increases in the number of antibiotic mixtures were accompanied by coordinated increases in multiple resistance classes. The eigenvalues, percentage of variance explained by each principal component, and loadings for all antibiotic and resistance variables are summarized in Supplementary Table S5.

The PCA linking antibiotic exposure at time t − 1 with resistance measured at time t (Figure 7B) demonstrated consistent carryover effects. Fluoroquinolones at t − 1 strongly aligned with CIP_Resis._ at t, whereas carbapenems and β-lactams at t − 1 aligned with CARB_Resist._ and CTG_Resis._ at t. MDR% occupied an intermediate position, reflecting the integration of multiple antibiotic pressures. These patterns confirm the temporal coupling between antibiotic inputs and subsequent shifts in resistance profiles.

When PCA was performed using crop type and fertilization regime (Figure 7C), clear clustering was observed. Soybean and maize rhizosphere soils were separated from no-plant soils along PC1 and PC2, indicating that plant presence altered the antibiotic–resistance relationships. Resistance vectors (especially CARB_Resis._) and (CIP_Resis._) shifted toward planted soils, suggesting that rhizosphere activity enhances antibiotic degradation gradients or selective microenvironments.

Resistance-only PCA (Figure 7D) further showed that rhizosphere soils exhibited higher multiresistance scores than no-plant soils. The soybean and maize soils clustered closer to CARB_Resis._ and CIP_Resis._ vectors, whereas no-plant soils occupied the lower-resistance quadrants. This reinforces the notion that vegetation, rather than fertilization alone, plays a central role in structuring resistance in amended soils.

## 3. Discussion

This study demonstrates that, under the tested application rate, sewage sludge (biosolids) functions as a safe and efficient fertilizer for soybean and maize, enhancing plant nutrition and biomass while accelerating the dissipation of antibiotic residues in the soil. Importantly, no antibiotic residues or phenotypic resistance were detected in the grains, confirming the food safety of biosolid fertilization under the conditions studied. The antibiotic residues introduced by biosolid amendment were mainly fluoroquinolones and tetracyclines, which declined progressively during cultivation. The faster decline observed in planted soils relative to bulk controls supports the idea that rhizosphere activity enhances contaminant attenuation through microbial degradation, root exudation, and plant uptake [22,23]. Between crops, maize soils often reached non-detectable levels earlier than soybean soils, likely reflecting crop-specific differences in rhizosphere microbiome composition, transpiration fluxes, and root architecture, which modulate contaminant turnover [24,25,26].

From an agronomic perspective, biosolids improved N, P, and K uptake, as well as total biomass in both crops, consistent with their recognized value as slow-release nutrient sources. These benefits occurred without impairing soybean nodulation or nitrogenase activity, suggesting that the antibiotic levels in the soil were below the ecotoxicological thresholds for *Bradyrhizobium* symbiosis [19,27,28]. Thus, the concentrations detected in biosolid-amended soils were insufficient to disrupt symbiotic functioning or induce measurable phytotoxicity, reinforcing the compatibility of biosolid fertilization with sustainable legume cultivation and its role in circular nutrient-recycling.

Regarding microbial dynamics, ARB were detected in all soils, including mineral and plant-free treatments, indicating a natural background resistome rather than contamination derived exclusively from biosolids. Ciprofloxacin-resistant isolates were detected in one single sampling point, even in the presence of fluoroquinolone residues, a pattern consistent with the generally low bioavailability and strong sorption of fluoroquinolones in the soil [29]. Resistance to third-generation cephalosporins and carbapenems was frequent across samples; however, the levels remained low, highly variable, and did not follow a directional trend associated with biosolid application. These findings are consistent with evidence that β-lactam resistance is widespread in environmental microbiomes and is not necessarily linked to anthropogenic inputs [30].

During early incubation (15 d), higher proportions of MDR isolates were observed under sludge fertilization; however, these peaks were transient and declined sharply by mid- and late-season sampling. By day 90, resistance levels converged across treatments, returned to baseline values, and showed no persistent amplification in biosolid-amended soils. This suggests that competitive interactions, redox fluctuations, and microbial turnover in the rhizosphere may suppress the long-term survival of resistant strains [31]. Therefore, the short-term increases in resistance frequency reflect ecological fluctuations within the soil microbial community rather than biosolid-driven selection. Therefore, short-term increases in resistance frequency reflect ecological fluctuations within the soil microbial community rather than biosolid-driven selection.

The multivariate analysis supported this interpretation. PCA revealed that crop presence, not fertilizer type, was the principal driver of sample ordination, indicating that rhizosphere processes dominated resistance dynamics. Antibiotic concentration gradients exerted only weak structuring effects, and the alignment between antibiotic classes and resistance endpoints was modest in this study. This finding suggests that biosolid-derived antibiotics were insufficient to impose selective pressure capable of reshaping the resistance structure, consistent with prior studies showing that environmental concentrations often fall below the minimum selective concentration (MSC) for AMR [32]. Lagged PCA further indicated that antibiotic concentrations at time t − 1 had limited predictive power for resistance at time t, reinforcing the absence of directional selection. Crucially, bacterial isolates recovered from soybean and maize grains were susceptible to all the tested antibiotics, with MICs at or below the clinical breakpoints. No multidrug resistance was observed, and LC–MS/MS confirmed the absence of antibiotic residues in the grains. Therefore, the soil resistome did not translate into phenotypic resistance or chemical residues in edible plant compartments, supporting the food chain safety of biosolids used under regulated conditions.

Collectively, these findings confirm that biosolid fertilization does not compromise food safety. The observed resistance dynamics occurred within the soil microbial community and were transient, low in magnitude, and ecologically constrained by competition in the plant rhizosphere. When properly stabilized and applied at agronomically relevant rates, biosolids increased plant biomass and grain production without detectable transfer of antibiotics or phenotypically resistant bacteria into edible tissues, reinforcing their potential as a safe fertilizer in integrated nutrient management systems. The findings also have practical implications for biosolid management in tropical and subtropical agriculture, where nutrient recycling, soil restoration, and waste valorization are policy priorities. The absence of detectable increases in phenotypic antimicrobial resistance suggests that Class A biosolids, when correctly processed and regulated, can be compatible with crop production and food safety goals. Future investigations should expand these assessments to ARG-level responses, long-term soil microbiome dynamics, antibiotic transformation pathways, and multi-season field trials to improve our understanding of the AMR risks associated with biosolid reuse. Importantly, this study evaluated only culturable microorganisms and a limited suite of antibiotics. Antibiotic resistance genes (ARGs) were not quantified; therefore, latent genetic-level changes that do not manifest phenotypically cannot be fully excluded. Additionally, the short-term nature of greenhouse experiments does not capture multi-seasonal or long-term ecological dynamics. Within this methodological scope, no sustained increase in phenotypic resistance was observed; however, future genomic and metagenomic approaches are required to fully assess potential subtle or cumulative AMR shifts.

## 4. Regulatory Recommendations

Based on the combined chemical, microbiological, and multivariate evidence generated in this study, several regulatory recommendations can be proposed to strengthen the safe and sustainable use of biosolids in agriculture.

Stringent treatment and hygienization standards must be maintained. The absence of clinically relevant ARB and the rapid dissipation of antibiotic residues confirm that properly stabilized biosolids pose minimal risk when regulatory thresholds for pathogen reduction and organic matter stabilization are followed. Policies should continue to enforce Class A/Type 1 sanitization requirements, ensuring that sludge undergoes validated thermal, alkaline, and advanced biological treatment processes.

Routine monitoring of antibiotic residues and AMR markers should be implemented. Although resistance remained at background levels and ciprofloxacin-resistant isolates were isolated just in a sampling point, periodic monitoring of key sentinel antibiotics (e.g., fluoroquinolones and tetracyclines) and resistance indicators (CTG_Resis._, CARB_Resis._, CIP_Resis._, and MDR%) is recommended, particularly in regions with high sludge inputs or variable wastewater profiles. Monitoring should follow validated LC–MS/MS protocols compatible with ng·kg^−1^ quantification.

Adopt plant-based bioattenuation as a risk-mitigation strategy. The accelerated dissipation of antibiotics in planted soils demonstrates that crops provide measurable ecological benefits. Regulatory frameworks may incorporate vegetation-based attenuation (phytobiodegradation) as a recognized mitigation measure, particularly for pharmaceuticals known to persist in bare soil.

Standardized risk assessment models incorporating multivariate analyses should be promoted. Traditional single-parameter regulatory assessments often fail to capture the complexities of antibiotic mixture dynamics. Multivariate ordination approaches (e.g., PCA and CCA) demonstrate high sensitivity for detecting subtle antibiotic–resistance relationships and should be recommended as complementary tools for environmental AMR evaluations.

Emphasizing grain and food safety evaluation using MIC profiling. The complete absence of antibiotic residues and resistant bacteria in edible grains underscores the safety of regulated biosolid use in the food chain. Regulatory agencies should continue to require periodic MIC testing of crop-derived isolates following CLSI/EUCAST standards, particularly for crops intended for direct human consumption.

Incorporate One Health principles into biosolid regulations. Given that biosolids contribute simultaneously to nutrient cycling, soil health, and waste reduction, One Health-aligned policies should explicitly recognize that well-treated biosolids do not increase AMR risk, supporting their use within circular economy strategies. We encourage long-term field trials and surveillance frameworks. While greenhouse studies provide controlled assessments, regulators should incentivize multi-season field monitoring programs to assess AMR dynamics under variable climatic, edaphic, and cropping conditions to ensure robust long-term biosafety.

Clear communication guidelines should be developed for farmers and wastewater managers. To prevent misperceptions about AMR risks, regulatory agencies should provide transparent guidance indicating that sanitized biosolids applied at agronomic rates do not introduce or amplify antibiotic resistance, thereby reinforcing stakeholder confidence. Collectively, these recommendations support evidence-based regulations that maximize the agronomic and environmental benefits of biosolids while safeguarding public health.

## 5. Materials and Methods

### 5.1. Experimental Design and Greenhouse Conditions

The experiment was conducted in a greenhouse at the UFPR in Curitiba (Paraná, Brazil). The greenhouse maintained a natural photoperiod, with an average daily photosynthetically active radiation (PAR) of ~800 μmol photons m^−2^ s^−1^, temperature ranging from 18 to 30 °C, and relative humidity of 60–80%. Soil moisture was maintained at 65% of field capacity by automated irrigation, which is considered optimal for both soybean and maize growth. The pots were made of high-density polyethylene without drainage holes to prevent leaching. Each pot contained 8.8 kg of air-dried soil that was previously homogenized with the assigned fertilization treatment. For each crop (soybean and maize) and fertilization treatment (Mineral or Sludge), 15 pots per treatment were prepared. Rhizospheric soil and plants were sampled destructively at 15, 45, and 90 d after sowing (*n* = 5 per sampling). These time points were chosen because they coincide with critical developmental stages: the early vegetative phase (establishment of the soybean–*Bradyrhizobium* symbiosis and root system expansion in maize), reproductive onset (high metabolic demand and peak nutrient uptake), and physiological maturity (yield determination and final residue partitioning). In parallel, we established an additional set of plant-free microcosms (*n* = 15 pots; 5 per sampling at 15, 45, and 90 days) prepared and managed identically to the fertilization treatments, but without sowing. These plant-free pots were used to quantify the natural dissipation of antibiotics and background dynamics of antibiotic-resistant bacteria (ARB) in bulk soil, that is, in the absence of rhizosphere effects. To minimize external colonization and surface algal growth while preserving gas exchange, the pots were capped with an inert, permeable nylon mesh (100 μm) held by a ring frame. At each sampling time (15, 45, and 90 days), bulk soil (0–10 cm) was collected from the center of each pot using a sterile corer after removing the top 0.5 cm to avoid surface artifacts. Subsamples were allocated for antibiotic analysis (stored at −20 °C) and culture-based ARB quantification (processed immediately). This design allowed us to track the temporal dynamics of antibiotic residues, resistant bacteria, and crop performance across the growth cycle.

### 5.2. Soil and Sewage Sludge Source Characterization

Soil was collected from the UFPR-Canguiri farm, a site free of prior agrochemical use, ensuring negligible background contamination with pesticides and antibiotics. A composite sample was analyzed for its chemical and physical properties (Supplementary Table S6). The soil was air-dried, sieved to 4 mm, and homogenized prior to use. Sewage sludge was obtained from a municipal wastewater treatment plant in Curitiba which operates a conventional activated-sludge process followed by mechanical dewatering and alkaline stabilization. After dewatering, quicklime was added in a dedicated covered mixing unit until the sludge pH exceeded 12.0, and the material was stored in roofed concrete bays for at least several weeks before use. This sequence of biological treatment, mechanical dewatering, alkaline stabilization, and storage corresponds to the conventional stabilization and hygienization procedure employed by the local sanitation company to produce Class A biosolids in Brazil, in accordance with the criteria established by CONAMA Resolution 375/2006 [33]. The chemical and microbiological properties of the samples are shown in Supplementary Table S1. For chemical characterization, a subsample of the sewage sludge was air-dried, ground to pass a 2 mm sieve, and digested with a mixture of concentrated HNO_3_ and HClO_4_ (3:1, *v*/*v*/ Sigma-Aldrich, São Paulo, Brazil). Total N was determined by the Kjeldahl method, whereas P was quantified colorimetrically by the molybdenum-blue method after acid digestion and K, Ca, Mg, Cu, Zn, Ni, Cr, and Pb were measured by inductively coupled plasma optical emission spectrometry (ICP–OES; model 720-ES, Varian, Inc., Palo Alto, CA, USA). Antibiotic concentrations in the biosolids were quantified using the same LC–MS/MS method described for soil samples (Section 5.5), with extraction based on the procedure of Rocha et al. [34] optimized for solid environmental matrices and instrument parameters and validation data summarized in Supplementary Table S7.

Application rates were calculated based on the K requirement of soybean and maize, as defined by the Paraná fertilization and liming guidelines [35]. For soybean, the recommended K_2_O inputs are 80–100 kg ha^−1^, and for maize, 90–120 kg ha^−1^. Because using nitrogen or phosphorus as the reference nutrient would have resulted in unrealistically low application rates that impaired homogenization, potassium was chosen as the reference element. Based on this criterion, 200 g of dry sludge was incorporated into 8.8 kg of soil per pot, equivalent to approximately 59 t ha^−1^ (0–20 cm, ρ = 1.3 g cm^−3^). This dose delivered a total of 2.899 g N, 1.392 g P, and 0.141 g K per pot (equivalent to 855.2 kg N ha^−1^, 410.7 kg P ha^−1^, and 41.68 kg K ha^−1^). When expressed as oxides, the applied amounts corresponded to 940.9 kg P_2_O_5_ ha^−1^ and 50.22 kg K_2_O ha^−1^. In the Mineral treatment, equivalent amounts of nutrients were supplied using analytical-grade salts. Nitrogen was supplied as urea [CO(NH_2_)_2_] (Sigma-Aldrich, São Paulo, Brazil), phosphorus as phosphoric acid (H_3_PO_4_, 85% *w*/*w*, Sigma-Aldrich, São Paulo, Brazil), and potassium as potassium sulfate (K_2_SO_4_, Sigma-Aldrich, São Paulo, Brazil). All salts were dissolved in ultrapure water before application, and the same volume of ultrapure water was used for sludge treatment as a blank control.

Following fertilization, the soil was thoroughly homogenized to ensure a uniform distribution of nutrients and organic matter. The pH was adjusted to 6.2 ± 0.1 (measured in H_2_O), corresponding to 5.6–5.7 in 0.01 M CaCl_2_, which is suitable for both soybean symbiotic performance and maize growth. Adjustments were carried out using finely ground dolomitic limestone [CaMg(CO_3_)_2_] (Sigma-Aldrich, São Paulo, Brazil). When minor downward corrections were necessary, dilute H_2_SO_4_ (0.05 mol L^−1^, Sigma-Aldrich, São Paulo, Brazil) was carefully incorporated into the moist soil. The treated soils were incubated for 10–14 days at 25 ± 2 °C under 60% water-holding capacity, with periodic mixing to promote aeration. During equilibration, the soil pH and electrical conductivity were verified to confirm stability prior to planting. All procedures complied with the Brazilian legislation on biosolid use in agriculture [33].

### 5.3. Plant Material and Evaluations

Soybean (*Glycine max*) seeds were inoculated with the commercial formulation Total Nitro Metabolic HC (Biotrop, Vinhedo, Brazil), containing strains SEMIA 5079 and SEMIA 5080 at a concentration of 7 × 10^9^ CFU mL^−1^, following the manufacturer’s recommendation of 2 mL per kg of seeds. The inoculant was homogenized with the seeds immediately prior to sowing. Maize (*Zea mays*) seeds were sown without any inoculation. Three seeds were initially planted per pot, and the plants were thinned to one uniform seedling per pot after emergence. Plants were cultivated until they reached physiological maturity.

At each harvest, plant samples (roots, leaves, and grains at 90 days) were collected for antibiotic quantification, nutrient content (N, P, and K), and microbiological analyses. Fresh biomass was recorded immediately after harvest, and dry biomass was determined after oven-drying at 65 °C to a constant weight. For leaves, samples were standardized as follows: in soybean, the first fully expanded, physiologically mature leaf was collected; in maize, the leaf immediately below the ear (flag leaf or adjacent) was collected. At 45 days (V4 stage), nodules from soybean roots were detached, counted, classified by diameter (≤2 mm, 2–4 mm, and >4 mm), and weighed. Their nitrogenase activity was assessed using the acetylene reduction assay (ARA) [19]. The effective nodulation index was determined based on the percentage of pink nodules (indicative of leghemoglobin activity). For N, P, and K analyses, plant material was dried at 65 °C to a constant mass, finely ground, and analyzed following standard protocols recommended by the Instituto de Desenvolvimento Rural do Paraná [35]. Total nitrogen was determined using the Kjeldahl method, phosphorus content was quantified calorimetrically using the molybdenum blue method after acid digestion, and potassium was measured using flame photometry. The results are expressed on a dry mass basis.

### 5.4. Antibiotics and Antibiotic-Resistant Bacteria Evaluations

Antibiotic residues were quantified in both the soil and plant tissue samples. At each sampling time (15, 45, and 90 days), bulk soil (10 g, 0–10 cm) was collected from the center of each pot using a sterile corer after removing the top 0.5 cm to avoid any surface artifacts. Subsamples were allocated for antibiotic analysis (stored at −20 °C) and culture-based ARB quantification (processed immediately). For ARB isolation, 25 g of sludge, soil, and fertilized soil samples, along with 1 g of seeds, were macerated using a sterile support with 225 mL of TSB broth (Sigma-Aldrich, São Paulo, Brazil) in a borosilicate bottle. Additionally, 500 mL of water was filtered through a 0.45 µm membrane, and the membrane was immediately added to a sterile 50 mL Falcon tube containing 15 mL of TSB broth. The bottles were incubated for 4 h at 35 ± 2 °C. Subsequently, 100 µL of each sample was transferred to three microtubes containing 900 µL of TSB broth (Tryptic Soy Broth—Oxoid, Basingstoke, England) along with the following antimicrobials: 1. Ceftriaxone—2 µg/mL, 2. Meropenem—2 µg/mL, and 3. Ciprofloxacin—0.5 µg/mL (all from Sigma-Aldrich, São Paulo, Brazil). The tubes containing the samples were incubated under selective pressure for 24 h at 35 ± 2 °C. After this period, 100 µL was seeded onto chromogenic agar (Himedia, Mumbai, India) with the addition of the same antimicrobials and concentrations. After incubation for 18 h at 35 ± 2 °C, the isolated microorganisms were identified using the Bruker (Billerica, MA, USA) MALDI Biotyper and subjected to antimicrobial susceptibility testing by agar microdilution, performed according to CLSI guidelines.

Antibiotics form soil and sludge samples were extracted according to the protocol of Rocha et al. [34], which was optimized for solid environmental matrices, whereas roots, shoots, and grains were extracted following the method of Marques et al. [36], which was validated for the recovery of pharmaceuticals in plant tissues. All extracts were analyzed using an LC–MS/MS system comprising a Xevo TQD triple-quadrupole mass spectrometer (Waters Corporation, Milford, MA, USA) equipped with an electrospray ionization (ESI) source, coupled to an HPLC system (model SYSLC-240-E, Varian Inc., Palo Alto, CA, USA) with an autosampler. Quantification was performed using deuterated internal standards (Sigma-Aldrich, São Paulo, Brazil), and the instrument parameters were optimized for each compound. Analytical validation included the determination of linearity, limits of detection (LOD), limits of quantification (LOQ), recovery efficiency, and matrix effects. The full chromatographic parameters and validation data are presented in Supplementary Table S7. Quality assurance included procedural blanks and duplicate analysis.

ARB were isolated from rhizospheric soil and plant tissues using a selective culture procedure [37]. Homogenized samples were plated on agar media supplemented with sentinel antibiotics, including ciprofloxacin, ceftriaxone and meropenem at clinically relevant breakpoint concentrations. After incubation, all different bacterial colony were isolated and stored. Isolates were identified at the species or genera level using the MALDI-TOF mass spectrometry (MALDI Biotyper, Bruker Daltonics, Bremen, Germany) and the antimicrobial susceptibility were evaluated using agar microdilution method [38].

Bulk soil from plant-free microcosms was processed in parallel using the same procedure, enabling direct comparison between rhizosphere and background dynamics. Multidrug resistance (MDR) was defined as resistance to at least one agent in two or more different antibiotic classes, following the criteria described by Hawser et al. [39]. MDR percentages were calculated as the proportion of isolates fulfilling this criterion relative to the total number of resistant isolates per sample.

### 5.5. Statistical Analyses

The planted experiment followed a two-factor design (Crop × Fertilization) with *n* = 5 pots per time; data normality and homoscedasticity were checked before two-way ANOVA and Tukey’s HSD (*p* < 0.05). GLMMs (binomial) were used to compare the proportions of pink nodules. Non-inferiority tests (margin −10%) evaluated whether sludge was not inferior to mineral fertilization for the agronomic endpoints. To quantify the effects of plants on antibiotics and ARB under sludge amendment, we compared rhizosphere soils (soybean and maize, sludge treatment) with plant-free bulk soil microcosms at matched times (15, 45, and 90 days). For antibiotic concentrations (log_10_-transformed when needed) and CFU counts (log_10_-transformed), we fitted linear mixed-effects models with fixed effects of plant presence (yes/no), time, and their interaction, and random intercept for pot. When appropriate, analyses were stratified by crop to contrast the soybean vs. maize rhizosphere against bulk soil. MDR proportions were analyzed using GLMMs (binomial or beta regression when justified). *p*-values were adjusted for multiple comparisons using the Benjamini–Hochberg test.

Multivariate analyses were performed to explore joint patterns between soil antibiotic concentrations and bacterial resistance endpoints. All multivariate procedures were conducted in Python (v.3.11) using scikit-learn. Prior to analysis, variables were standardized using z-score normalization to ensure comparable scale across antibiotics and resistance metrics. A Principal Component Analysis (PCA) integrating all antibiotic concentrations and resistance endpoints (CTG_Resis._, CARB_Resis._, CIP_Resis._, MDR%) was first conducted to evaluate global patterns across the experiment. A second PCA incorporating lagged variables was performed by pairing antibiotic concentrations at time t − 1 (0, 15, and 45 days) with resistance endpoints measured at the subsequent sampling time t (15, 45, and 90 days), allowing identification of predictive associations between prior antibiotic exposure and subsequent resistance. A third PCA included crop type (soybean, maize, or no-plant) and fertilization regime (sludge vs. mineral) as grouping factors, while retaining antibiotic and resistance variables to evaluate vegetation effects on antibiotic–resistance structuring. A fourth PCA used only resistance endpoints to visualize changes in resistance patterns driven by vegetation and fertilization. All biplots were generated using type-2 scaling (scores vs. loadings × √eigenvalue), enabling simultaneous interpretation of sample ordination and variable contributions.

## 6. Conclusions

This study demonstrates that the application of properly treated biosolids at agronomic rates is both safe and effective for soybean and maize cultivation in the subtropical region of southern Brazil, specifically in the Curitiba area (Paraná State). Biosolids improved plant nutrition and biomass accumulation without impairing nodulation or crop performance. Antibiotic residues, initially present in the biosolid-amended soils, declined steadily over time and reached non-detectable levels in all planted treatments by 90 days, confirming active rhizosphere-driven dissipation. The updated antibiotic resistance dataset further indicates that biosolids do not introduce or amplify clinically relevant antimicrobial resistance. ARB were detected across all soils, including mineral-fertilized and plant-free controls, highlighting the presence of natural background resistome rather than biosolid-mediated contamination. Ciprofloxacin-resistant isolates were recovered in just one sampling point, and resistance to carbapenems and third-generation cephalosporins remained low and ecologically variable, returning to the background levels by the end of the experiment. Multivariate analyses (PCA) integrating all updated variables revealed that plant presence, rather than biosolid fertilization, structured soil microbial patterns, and antibiotic concentration gradients exhibited weak correspondence with resistance endpoints, providing no evidence of selective amplification. Crucially, no antibiotic residues or resistant bacteria were detected in soybean or maize grains, and all isolates displayed MIC values below the CLSI/EUCAST clinical thresholds, confirming that neither antibiotic residues nor resistance traits were transferred to edible tissues. Taken together, the chemical, microbiological, and multivariate evidence demonstrates that under the conditions tested, biosolids application did not result in measurable increases in phenotypic antibiotic resistance. These findings reinforce that regulated, sanitized biosolids represent a sustainable and One Health-compatible fertilization strategy, capable of enhancing agricultural productivity while maintaining environmental and public health safeguards. The results strongly support the continued and expanded use of biosolids in integrated nutrient management programs, provided that the treatment and monitoring standards are maintained.

## Figures and Tables

**Table 1:** Abundance of antibiotic-resistant bacteria (ARB) isolated from soybean and maize rhizospheres under mineral or sludge fertilization, and from bulk soil without plants, across sampling times (0, 15, 45, and 90 days). The number of resistant isolates to third-generation cephalosporins (CTG), carbapenems (CARB), and ciprofloxacin (CIPRO), and the proportion of multidrug-resistant isolates (MDR) are shown.

Days	Plant	Treatment	CTG_Resis._	CARB_Resis._	CIPRO_Resis._	MDR	%MDR
0	Corn	Chemical fertilizer	1.00	0.67	0.00	0.00	0.00
		Sewage sludge	1.00	0.67	0.00	0.00	0.00
	Soybean	Chemical fertilizer	1.00	0.67	0.00	0.00	0.00
		Sewage sludge	1.00	0.67	0.00	0.00	0.00
	No plant	Chemical fertilizer	1.00	0.67	0.00	0.00	0.00
		Sewage sludge	1.00	0.67	0.00	0.00	0.00
15	Corn	Chemical fertilizer	1.00	1.00	0.00	0.00	0.00
		Sewage sludge	0.50	0.75	0.25	0.25	25.00
	Soybean	Chemical fertilizer	0.67	0.67	0.67	0.67	67.00
		Sewage sludge	0.67	0.67	0.00	0.00	0.00
	No plant	Chemical fertilizer	0.40	0.40	0.20	0.20	20
		Sewage sludge	0.00	1.00	0.00	0.00	0.00
45	Corn	Chemical fertilizer	0.00	1.00	0.00	0.00	0.00
		Sewage sludge	1.00	0.00	0.00	0.00	0.00
	Soybean	Chemical fertilizer	0.33	0.67	0.00	0.00	0.00
		Sewage sludge	0.00	1.00	0.00	0.00	0.00
	No plant	Chemical fertilizer	0.50	0.50	0.00	0.00	0.00
		Sewage sludge	0.67	0.67	0.00	0.00	0.00
90	Corn	Chemical fertilizer	0.33	1.00	0.00	0.00	0.00
		Sewage sludge	1.00	1.00	0.00	0.00	0.00
	Soybean	Chemical fertilizer	0.33	0.67	0.00	0.00	0.00
		Sewage sludge	0.50	0.50	0.00	0.00	0.00
	No plant	Chemical fertilizer	0.00	1.00	0.00	0.00	0.00
		Sewage sludge	1.00	0.67	0.00	0.00	0.00

MDR was defined as resistance to ≥2 antibiotics. The %MDR was calculated as the proportion of MDR isolates relative to the total number of resistant isolates at each treatment and sampling time.

## Data Availability

All data supporting the findings of this study are available from the corresponding author upon reasonable request.

## Supplementary Materials

The following supporting information can be downloaded at: https://www.mdpi.com/article/10.3390/antibiotics14121244/s1, Table S1: Physicochemical properties of the sewage sludge; Table S2: Soybean nodulation parameters at 45 days under mineral and sludge fertilization; Table S3: Two-way ANOVA summary (F-values and significance levels) for nutrient concentrations in soybean and corn; Table S4: Identity and abundance of antibiotic-resistant bacteria (ARB) isolated from soybean and maize rhizospheres, and from bulk soil without plants, under mineral or sludge fertilization across sampling times (0, 15, 45, and 90 days); Table S5: Summary of multivariate analyses (PCA/CCA) integrating antibiotic concentrations and bacterial resistance endpoints; Table S6: Physicochemical properties of the experimental soil (UFPR-Canguiri farm); Table S7: LC–MS/MS parameters for antibiotics analyzed in soil, sludge, and plant samples.
