# Supplementary material for: Biosolids as Safe Fertilizers for Soybean and Maize: Enhanced Nutrition Without Antibiotic Residues or Phenotypic Resistance in Grains
Source: Antibiotics (Basel). 2025 Dec 9;14(12):1244. doi: 10.3390/antibiotics14121244 (PMC12729575; doi:10.3390/antibiotics14121244)
Supplement: Supplementary file 1 [file antibiotics-14-01244-s001.zip › antibiotics-4023644-supplementary.pdf]

antibiotics

Article

Biosolids as Safe Fertilizers for Soybean and Maize: Enhanced Nutrition Without Antibiotic Residues or Phenotypic Resistance in Grains

Thiago Nery de Menezes [1,2], Keite da Silva Nogueira [1,2], Ruanita Veiga Queiroz Apolinário [1,2], Raizza Zorman Marques [3], André Carlos Auler [4], Leandro Flávio Carneiro [5], Murilo Duma [6], Rebert Skalisz [6], Marcelo Pedrosa Gomes [3,*]

1 Departamento de Patologia Básica, Setor de Ciências Biológicas, Universidade Federal do Paraná, Avenida Coronel Francisco H. dos Santos, 100, Centro Politécnico Jardim das Américas, CEP 19031, 81531-980, Curitiba, Paraná, Brazil.
2 Laboratório de Bacteriologia, Complexo Hospital de Clínicas, Universidade Federal do Paraná, Rua Padre Camargo, 280, CEP 80060-240, Curitiba, Paraná, Brazil; keite.nogueira@ufpr.br.
3 Laboratório de Fisiologia de Plantas sob Estresse, Departamento de Botânica, Setor de Ciências Biológicas, Universidade Federal do Paraná, Avenida Coronel Francisco H. dos Santos, 100, Centro Politécnico Jardim das Américas, C.P. 19031, 81531-980, Curitiba, Paraná, Brazil.
4 Departamento de Solos e Engenharia Agrícola, Universidade Federal do Paraná, Rua dos Funcionários, 1540, CEP 80035-050, Curitiba, Paraná, Brazil.
5 Departamento de Fitotecnia e Fitosanidade, Universidade Federal do Paraná, Rua dos Funcionários, 1540, CEP 80035-050, Curitiba, Paraná, Brazil.
6 Companhia de Saneamento do Paraná (SANEPAR) - Sede Administrativa, Rua Engenheiros Rebouças, 1376, Rebouças, CEP 80215-900, Curitiba, Paraná, Brazil

* Correspondence: marcelo.gomes@ufpr.br

 https://doi.org/10.3390/xxxxx

Table S1. Physicochemical properties of the sewage sludge.

**Physicochemical properties**	**Value (mg/kg dry solids)**	**Macronutrients**	**Value (mg/kg dry solids)**	**Metals**	**Value (mg/kg dry solids)**	**Metals**	**Value (mg/kg dry solids)**	**Microbiological indicators**	**Value**
pH (H_2O)	12.3	Total nitrogen (N)	14,4945	Zinc (Zn)	1,677	Arsenic (As)	< LQM	Thermotolerant coliforms	< 0.54 NMP g^{-1} ST
Total solids (ST)	428.6	Total phosphorus (P)	6,960	Copper (Cu)	154.5	Cadmium (Cd)	< LQM	Viable helminth eggs (Ascaris)	< 0.1 eggs g^{-1} ST
Volatile solids (VS)	260.7	Total potassium (K)	706.4	Nickel (Ni)	41.7	Mercury (Hg)	< LQM		
Moisture content (%)	57.1	Total sulfur (S)	14,197	Chromium (Cr)	43.5	Molybdenum (Mo)	< LQM		
Organic carbon (TOC)	78.0	Calcium (Ca)	115,274	Lead (Pb)	21.4	Selenium (Se)	< LQM		
		Magnesium (Mg)	73,720	Barium (Ba)	245.1				

Table S2. Soybean nodulation parameters at 45 days.

Parameter	Mineral fertilization	Sludge fertilization
Nodules per plant (≤2 mm)	18 ± 4	20 ± 5
Nodules per plant (2–4 mm)	10 ± 3	11 ± 3
Nodules per plant (>4 mm)	6 ± 2	7 ± 2
Total nodules per plant	34 ± 6	38 ± 7
Fresh mass of nodules (g)	0.82 ± 0.12	0.88 ± 0.14
Dry mass of nodules (g)	0.26 ± 0.05	0.28 ± 0.06
Pink nodules (%)	72 ± 8	75 ± 9
Nitrogenase activity (μmol C_2H_4 g^{-1} h^{-1})	5.8 ± 1.0	6.1 ± 1.1

Table S3. Two-way ANOVA summary (F-values and significance levels) for nutrient concentrations in soybean and corn.

Soybean			
**Mineral**	**Treatment**	**DAP**	**Treatment × DAP**
N (%)	1244.81 ***	2920.0 ***	100.9 ***
P (%)	293.70 ***	382.8 ***	1.7
K(%)	1993.92 ***	1132.7 ***	160.7 ***
**Corn**			
N (%)	753.6 ***	4033.6 ***	0.62
P (%)	223.4 ***	363.3 ***	4.66 *
K(%)	2393.7 ***	913.7 ***	70.2 ***

* $p < 0.05$; ** $p < 0.01$; *** $p < 0.001$.

Table S4. Identity and abundance of antibiotic-resistant bacteria (ARB) isolated from soybean and maize rhizospheres, and from bulk soil without plants, under mineral or sludge fertilization across sampling times (0, 15, 45, and 90 days).

Days	Plant	Treatment	Microorganism
0	Corn	Chemical fertilizer	*Acinetobacter sp.*
			Pseudomonas chlororaphis
			Pseudomonas putida
		Sewage sludge	*Acinetobacter sp.*
			Pseudomonas chlororaphis
			Pseudomonas putida
	Soybean	Chemical fertilizer	*Acinetobacter sp.*
			Pseudomonas chlororaphis
			Pseudomonas putida
		Sewage sludge	*Acinetobacter sp.*
			Pseudomonas chlororaphis
			Pseudomonas putida
	No plant	Chemical fertilizer	*Acinetobacter sp.*
			Pseudomonas chlororaphis

 https://doi.org/10.3390/xxxxx

			Pseudomonas putida
		Sewage sludge	*Acinetobacter sp.*<br>*Pseudomonas chlororaphis*<br>*Pseudomonas putida*
15	Corn	Chemical fertilizer	*Pseudomonas putida*
		Sewage sludge	*Enterobacter bugandensis*<br>*Pseudomonas guariconensis*<br>*Pseudomonas monteilii*<br>*Pseudomonas putida*
	Soybean	Chemical fertilizer	*Acinetobacter calcoaceticus*<br>*Pseudomonas putida*<br>*Pseudomonas sp.*
		Sewage sludge	*Acinetobacter calcoaceticus*<br>*Pseudomonas monteilii*<br>*Pseudomonas putida*
	No plant	Chemical fertilizer	*Enterobacter bugandensis*<br>*Pseudomonas protegens*<br>*Pseudomonas putida*
		Sewage sludge	*Acinetobacter calcoaceticus*<br>*Enterobacter bugandensis*<br>*Klebsiella oxytoca*<br>*Pseudomonas guariconensis*<br>*Pseudomonas putida*
45	Corn	Chemical fertilizer	*Pseudomonas monteilii*<br>*Pseudomonas putida*
		Sewage sludge	*Pseudomonas monteilii*<br>*Pseudomonas putida*
	Soybean	Chemical fertilizer	*Enterobacter hormaechei*
		Sewage sludge	*Pseudomonas monteilii*<br>*Pseudomonas putida*<br>*Yokenella regensburgei*
	No plant	Chemical fertilizer	*Pseudomonas putida*
		Sewage sludge	*Enterobacter bugandensis*<br>*Pseudomonas monteilii*<br>*Pseudomonas putida*<br>*Yokenella regensburgei*
90	Corn	Chemical fertilizer	*Enterobacter bugandensis*<br>*Pseudomonas putida*<br>*Serratia marcescens*
		Sewage sludge	*Pseudomonas monteilii*<br>*Pseudomonas putida*<br>*Serratia marcescens*
	Soybean	Chemical fertilizer	*Pseudomonas putida*
		Sewage sludge	*Enterobacter asburiae*<br>*Pseudomonas monteilii*<br>*Pseudomonas putida*

No plant	Chemical fertilizer	*Enterobacter bugandensis*<br>*Serratia marcescens*
	Sewage sludge	*Pseudomonas putida*<br>*Pseudomonas monteilii*

Table S5. Summary of multivariate analyses (PCA/CCA) integrating antibiotic concentrations and bacterial resistance endpoints.

Analysis (Fig. 7)	Active variables	PC / Axis	Eigenvalue	Variance explained (%)	Cumulative variance (%)	Selected high loadings	Permutation test (p-value)
PCA – Panel A (antibiotics + resistance)	CIP, ENR, LEV, NOR, OTC, DOX, TC, SMX, SDZ, AMX, MER, CRO, GEN, CTGResis., CARBResis., CIPResis., MDR%	PC1	largest (dominant)	67.0%	67.0%	PC1 ↑: CIP, LEV, ENR, MDR%	— (PCA, no permutation)
		PC2	lower	8.7%	75.7%	PC2 ↑: CIPResis., MDR%	—
		PC3	smaller	7.3%	82.9%	PC3 ↑: CTGResis., CARBResis.	—
		PC4	small	4.9%	87.8%	—	—
PCA – Panel B (lag t–1 → t)	Antibiotics at t–1 + resistance at t	PC1	largest	~60–70%	~70%	PC1 ↑: fluoroquinolones (t–1), MDR% (t)	—
		PC2	moderate	~10–15%	~85%	PC2 ↑: CIPResis. (t), CEFT/Carbapenems (t–1)	—
PCA – Panel C (crop × fertilizer × all variables)	Same as Panel A	PC1	largest	identical to PCA-A	identical	PC1 ↑: antibiotics + MDR%	—
		PC2	identical	identical	identical	PC2 ↑: CIPResis., MDR%	—
PCA – Panel D (resistance only)	CTGResis., CARBResis., CIPResis., MDR%	PC1	largest	~70–75%	70–75%	PC1 ↑: CIPResis., MDR%	—
		PC2	smaller	~15–20%	~90%	PC2 ↑: CTGResis.	—

Table S6. Physicochemical properties of the experimental soil (UFPR-Canguiri farm).

Parameter	Value
pH (H_2O)	6.1
pH ($CaCl_2$)	5.5
Organic matter (%)	3.2
CEC (cmolc/kg)	11.8
Sand (%)	62
Silt (%)	18
Clay (%)	20
Total N (mg/kg)	980
Total P (mg/kg)	210
Total K (mg/kg)	145

Table S7. LC–MS/MS parameters for antibiotics analyzed in soil, sludge, and plant samples.

Antibiotic	Class	Precursor ion (m/z)	Product ions (m/z)	CE (eV)	RT (min)*	Internal standard
Ciprofloxacin	Quinolone	332.1	314.1; 231.0	20–30	3.2	Ciprofloxacin-d8
Enrofloxacin	Quinolone	360.2	342.1; 245.0	20–28	3.4	Ciprofloxacin-d8
Levofloxacin	Quinolone	362.1	318.0; 261.0	18–28	3.8	Ciprofloxacin-d8
Norfloxacin	Quinolone	320.1	302.0; 276.0	18–26	4.1	Ciprofloxacin-d8
Azithromycin	Macrolide	749.5	591.4; 158.1	25–35	6.7	Azithromycin-d3
Oxytetracycline	Tetracycline	461.1	426.1; 201.0	18–25	4.2	Tetracycline-d6
Doxycycline	Tetracycline	445.2	428.1; 154.0	20–28	4.5	Tetracycline-d6
Tetracycline	Tetracycline	445.2	410.1; 154.0	18–28	4.8	Tetracycline-d6
Sulfamethoxazole	Sulfonamide	254.0	156.0; 92.0	15–25	5.1	Sulfamethoxazole-d4
Sulfadiazine	Sulfonamide	251.0	108.0; 92.0	15–22	5.4	Sulfamethoxazole-d4
Amoxicillin	β-lactam	366.1	208.0; 114.0	15–25	2.1	—
Meropenem	β-lactam	384.1	141.0; 67.0	18–25	2.5	—
Ceftriaxone	β-lactam	555.1	396.0; 208.0	20–28	2.9	—
Gentamicin	Aminoglycoside	478.3 (B1), 450.3 (C1)	322.2; 160.1	20–30	1.8	—

Disclaimer/Publisher's Note: The statements, opinions and dataontained in all publications are solely those of the individual author(s) and contributor(s) and not of MDPI and/or the editor(s). MDPI and/or the editor(s) disclaim responsibility for any injury to people or property resulting from any ideas, methods, instructions or products referred to in the content.
